# Prognostic biomarker TUBA1C is correlated to immune cell infiltration in the tumor microenvironment of lung adenocarcinoma

**DOI:** 10.1186/s12935-021-01849-4

**Published:** 2021-03-02

**Authors:** Tingting Bian, Miaosen Zheng, Daishan Jiang, Jian Liu, Hui Sun, Xiaoli Li, Lei Liu, Jianguo Zhang, Yifei Liu

**Affiliations:** 1 Department of Pathology, Affiliated Hospital of Nantong University, Nantong, Jiangsu China; 2 School of Medicine, Nantong University, Nantong, Jiangsu China; 3 Department of Emergency Medicine, Affiliated Hospital of Nantong University, Nantong, Jiangsu China; 4 Department of Chemotherapy, Affiliated Hospital of Nantong University, Nantong, Jiangsu China

**Keywords:** TUBA1C, Lung adenocarcinoma, Tumor-infiltrating immune cells, Prognosis, Diagnosis

## Abstract

**Background:**

TUBA1C is a microtubule component that is involved in a variety of cancers. Our main objective was to investigate TUBA1C expression, its prognostic value, its potential biological functions, and its impact on the immune system of patients with lung adenocarcinoma (LUAD).

**Methods:**

The Cancer Genome Atlas (TCGA), Gene Expression Profiling Interactive Analysis (GEPIA) and Immunohistochemistry Analysis were used to analyze TUBA1C expression, its clinicopathology, overall survival (OS), and disease-free survival (DFS) in LUAD patients. We also determined the correlation between TUBA1C and tumor-infiltrating immune cells (TIICs) by using CIBERSORT and GEPIA databases. To determine the expression of TUBA1C in LUAD, we analyzed a collection of immune infiltration levels and cumulative survival of LUAD tissues in TIMER database. By using UALCAN, STRING, and GeneMANIA databases, we investigated the protein-coding genes related to TUBA1C and its co-expression genes in LUAD tissues. Gene set enrichment analysis (GSEA) was performed by using the TCGA dataset.

**Results:**

The mRNA and the protein expression of TUBA1C were found to be up-regulated in LUAD tissues. The univariate analysis indicated that an increased expression of TUBA1C was significantly correlated to the following parameters: age, stage, and lymph node metastasis. An over-expression of TUBA1C was associated with a poor prognosis of LUAD. In TIMER and CIBERSORT databases, we found that TUBA1C is correlated with 13 types of TIICs: activated B cell, activated CD4 T cell, central memory CD4 T cell, effector memory CD8 T cell, eosinophils, immature B cell, gamma-delta T cell, immature dendritic cell, mast cell, memory B cell, natural killer T cell, regulatory T cell, and type 2T helper cell. By performing GSEA, we found that TUBA1C is closely correlated to cell cycle, p53 signaling pathway, glycolysis, and gluconeogenesis.

**Conclusions:**

Our findings indicate that TUBA1C is associated with TIICs in tumor microenvironment. Therefore, it serves as a novel prognostic biomarker and a target for future treatment methods of LUAD.

**Supplementary Information:**

The online version contains supplementary material available at 10.1186/s12935-021-01849-4.

## Background

The morbidity and mortality of lung cancer has been the highest among all types of cancers, so it is the most common form of malignancy worldwide [1]. Lung adenocarcinoma (LUAD) is a major pathological subtype of non-small cell lung cancer (NSCLC), which approximately accounts for 40% of lung cancer cases [2]. Surgery, chemotherapy, radiotherapy, and targeted therapy are the conventional methods of treating patients with NSCLC; however, the prognosis of these patients has been unsatisfactory till date [3]. Since the past decade, immune checkpoint inhibitors (ICIS) have been used to treat patients with NSCLC, and they have modified the treatment pattern of this refractory disease [4]. Tumor-infiltrating immune cells (TIICs) have impacted the immune system and tackled abnormal biological behaviors in a complex way, so they play a key role in eliciting the body’s response to immunotherapy [5].

Microtubule is an important component of the eukaryotic cytoskeleton. Moreover, it is one of the most functional proteins, which play an important role in dynamic polymerization and depolymerization through cell replication and division [6]. Microtubules are uniformly assembled from highly conserved α/β-tubulin heterodimers [7]. Recently, several studies have reported that α-tubulin is involved in the occurrence of a variety of tumors, such as lung cancer, breast cancer and prostatic cancer [8–10]. In addition, α-tubulin is also associated with the development of astrocytoma and chemotherapeutic resistance of liver cancer [11]. Moreover, TUBA1C is a subtype of α-tubulin, and its overexpression is associated with the poor prognosis of hepatocellular carcinoma (HCC) and pancreatic ductal adenocarcinoma [12, 13]. However, no previous study has elucidated how the overexpression of TUBA1C affects LUAD patients.

In this study, IHC and Gene Expression Profiling Interactive Analysis (GEPIA) were performed to elucidate the correlation between TUBA1C and LUAD. Furthermore, we used the computational algorithms CIBERSORT and TIMER to explore the relationship between TUBA1C and TIICs in LUAD patients. In addition, STRING software, GeneMANIA Analysis, and Gene Set Enrichment Analysis (GSEA) were used to further study the function and mechanism of TUBA1C in LUAD patients. The findings proved that TUBA1C played an important role in the development of LUAD. Furthermore, we elucidated the potential relationship between TUBA1C and tumor-immune interaction.

## Materials and methods

### Data acquisition

The TCGA database (https://portal.gdc.cancer.gov/) was used to obtain the data of TUBA1C expression in LUAD tissues and normal tissues. A total of 535 tumor tissues (Additional file 1) and 59 normal tissues were included in the data analysis. Meanwhile, 390 LUAD tissues and 60 normal tissues were treated from the Affiliated Hospital of Nantong University in China. All patients were treated by surgical resection between 2012 and 2013. All clinical data on the patients were carefully recorded after the diagnosis of LUAD by two pathologists. The follow up was 60 months. All experiments involving patient specimens were approved by the Ethics Committee of the Affiliated Hospital of Nantong University, China.

### GEPIA analysis

Gene Expression Profiling Interactive Analysis (http://gepia.cancer-pku.cn/index.html) is an online database developed by scientists of Peking University, China [14]. To determine the expression of TUBA1C in LUAD tissues, we performed GEPIA and constructed boxplot, pathological stage plot, and survival curves, such as overall survival (OS) curve and disease-free survival (DFS) curve. Meanwhile, the relationship between TUBA1C and immune infiltrating cells was analyzed by GEPIA. The Spearman method was used to determine the correlation coefficient of the relationship.

### Immunohistochemistry analysis

An immunohistochemistry (IHC) assay was conducted as previously described [15]. Briefly, the LUAD samples were deparaffinized and rehydrated. The primary antibody was that against TUBA1C (1:100 dilution; ab222849; abcam). The positive expression of TUBA1C was localized in cytoplasm. The scoring criteria for IHC staining were based on the intensity of the stain and the percentage of immunoreactive cells, as previously described [15].

### TIMER analysis

In this experiment, we used TIMER (https://cistrome.shinyapps.io/timer/) algorithm to comprehensively elucidate the correlation between different types of tumors and TIICs [16]. Moreover, we performed a series of analyses to determine the expression of TUBA1C in different types of cancer and to comprehend its correlation with the abundance of TIICs. These TIICs were further classified as follows: B cells, CD4+ T cells, CD8+ T cells, macrophages, neutrophils, and dendritic cells. By using the TIMER algorithm, we performed a correlation analysis between TUBA1C, its related genes, and the markers of immune cells [17]. The expression of TUBA1C gene was plotted on the x-axis, and the expression of its related marker genes was plotted on the y-axis.

### CIBERSORT analysis

The computational method of CIBERSORT [18] (http://cibersort.stanford.edu/) is a deconvolution algorithm based on gene expression, and it is used to evaluate the changes of a group of genes relative to all other genes in a sample. Based on the expression of TUBA1C, we classified 497 samples into high and low expression groups. Using CIBERSORT algorithm, we measured the immune responses of 28 TIICs and evaluated the relationship between these TIICs and the expression of TUBA1C in LUAD tissues. Our main goal was to determine the correlation between these TIICs.

### UALCAN analysis

The University of Alabama Cancer Database (UALCAN) (http://ualcan.path.uab.edu/index.html) is a visual portal of the Cancer Genome Atlas (TCGA) database [19]. This database was used to analyze the positively and negatively expressed protein-coding genes related to TUBA1C in LUAD tissues.

### STRING and GeneMANIA analysis

To obtain the information on protein–protein interaction, most scientists prefer using the following two datasets: STRING and GeneMANIA [20, 21]. The protein–protein interaction network of TUBA1C was predicted with the help of STRING and GeneMANIA datasets.

### Gene set enrichment analysis (GSEA)

Gene set enrichment analysis (GSEA) was performed on the normalized RNA-Seq data, which was obtained from TCGA database [22]. The gene ontology (GO) terms and the Kyoto Encyclopedia of Genes and Genomes (KEGG) pathways were used to investigate the possible biological functions of TUBA1C. A false discovery rate (FDR) < 0.050 and a nominal P < 0.050 were considered to be statistically significant.

### Statistical analysis

All statistical analyses were performed by using R language (version 3.5.3). Kaplan–Meier univariate analysis was used to determine the effect of TUBA1C expression on survival. Multivariate Cox analysis was performed to determine the expression of TUBA1C and the effect of other pathological and clinical factors (age, gender, stage, tumor status, and lymph node) on overall survival (OS). The results were considered to be statistically significant when P < 0.05.

## Results

### The mRNA expression of TUBA1C in different tumors

**Fig.1:**
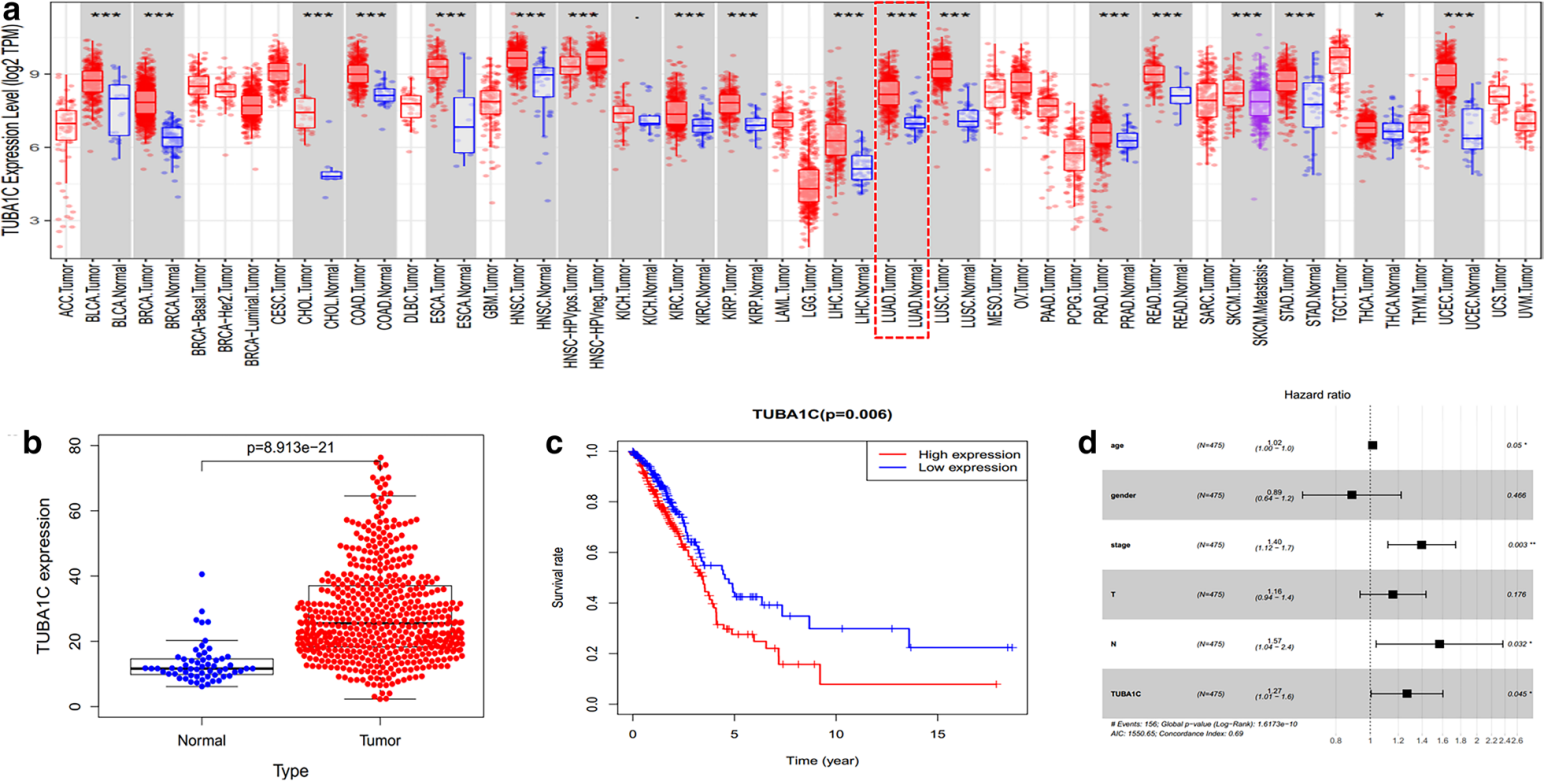
TUBA1C expression levels according to TCGA database. **a** TUBA1C expression levels in different tumor types from TCGA database were determined by TIMER (*P < 0.05, **P < 0.01, ***P < 0.001). **b** The expression of TUBA1C between normal and lung adenocarcinoma tissues. **c** Survival curve of differential TUBA1C expression in LUAD. **d** Multivariate Cox analysis of TUBA1C expression and other clinicopathological variables

In order to determine the difference between the expression of TUBA1C in tumor tissues and normal tissues, we used TIMER algorithm to analyze the mRNA expression of TUBA1C in different types of tumors that were obtained from TCGA database (Fig. 1a). Compared to normal tissues, the mRNA expression of TUBA1C was found to be higher in the following types of tumors: bladder urothelial carcinoma (BLCA), breast invasive carcinoma (BRCA), cholangiocarcinoma (CHOL), colon adenocarcinoma (COAD), esophageal carcinoma (ESCA), head and neck squamous cell carcinoma (HWSC), kidney renal clear cell carcinoma (KIRC), liver hepatocellular carcinoma (LIHC), LUAD, lung squamous cell carcinoma (LUSC), prostate adenocarcinoma (PRAD), rectum adenocarcinoma (READ), stomach adenocarcinoma (STAD), thyroid carcinoma (THCA), and uterine corpus endometrial carcinoma (UCEC).

### The levels of TUBA1C predicts the prognosis of LUAD

**Fig.2:**
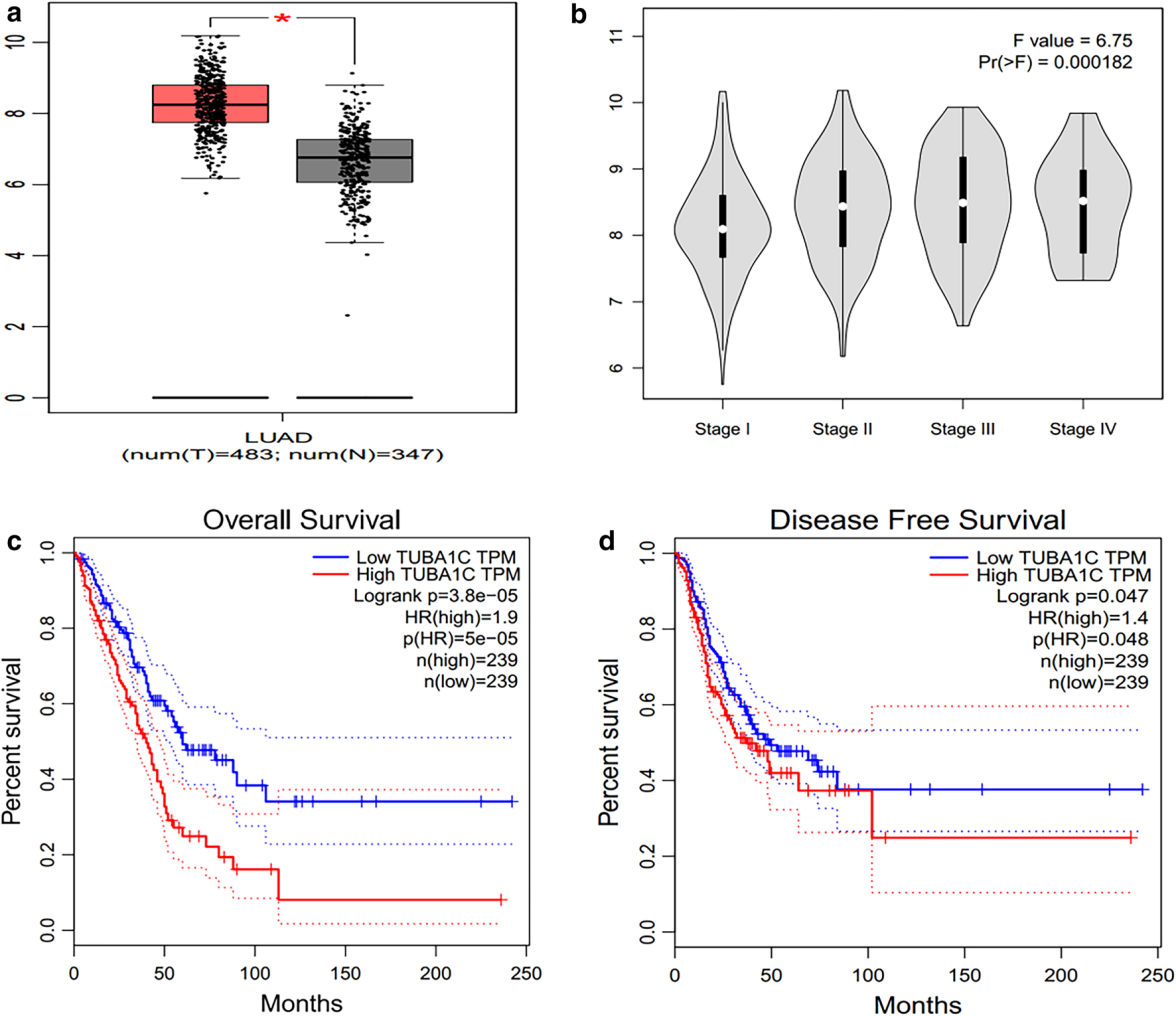
TUBA1C expression levels in LUAD performed by GEPIA. **a** TUBA1C mRNA expression levels in normal and LUAD tissues. **b** Differential expression of TUBA1C in different cancer grade. **c**, **d** Levels of TUBA1C mRNA expression, overall survival and disease-free survival based on data obtained from GEPIA

**Fig.3:**
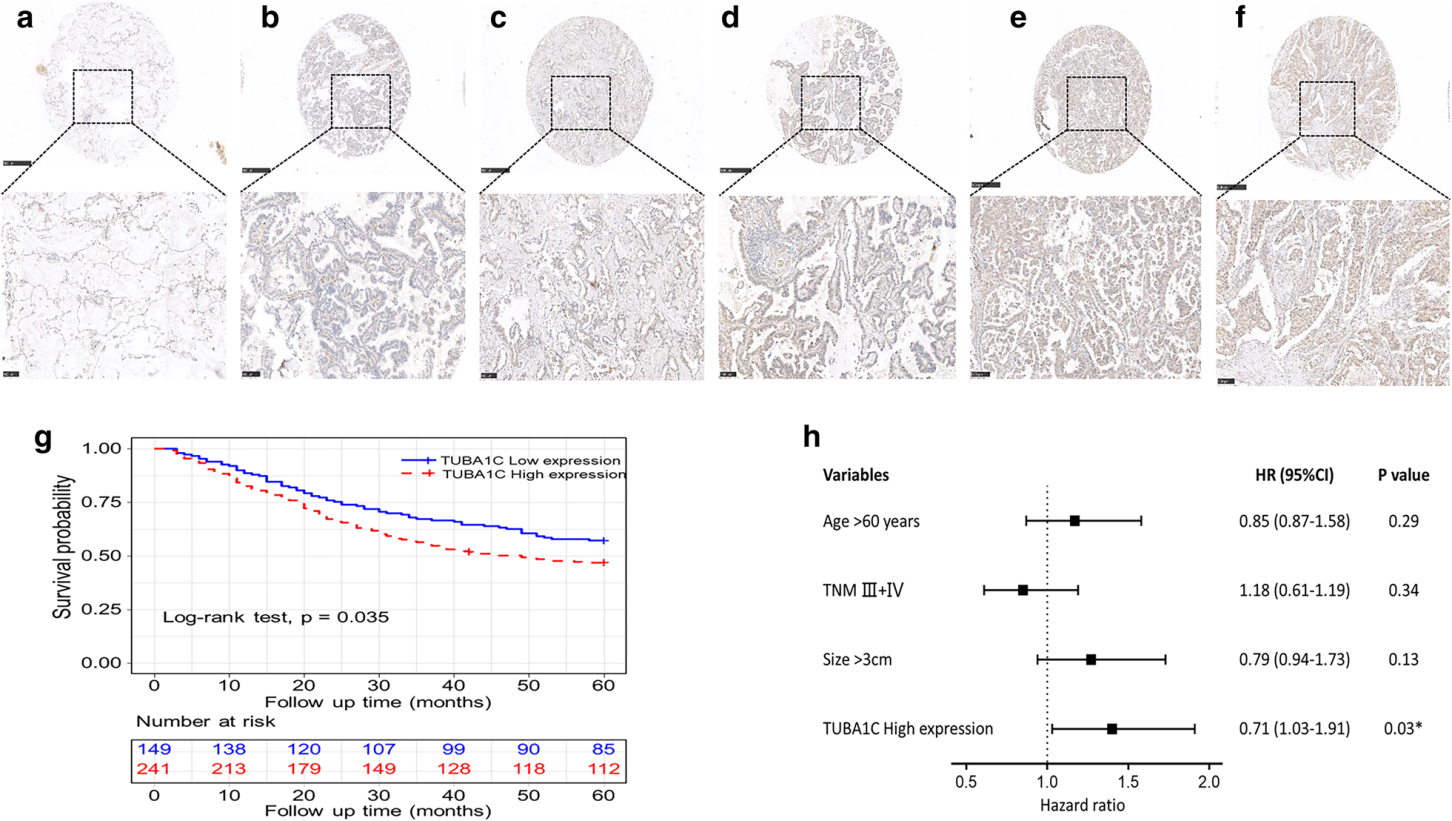
TUBA1C expression levels in LUAD performed by immunohistochemistry. **a–f** TUBA1C protein was detected in normal lung tissues and LUAD tissues by TMA-IHC. × 40 magnification (bar = 500 μm), × 200 magnification (bar = 100 μm). TUBA1C protein was dyed brown particles in cell cytoplasm. **g** TUBA1C overexpression was negatively associated with the overall survival rate of patients by Kaplan‑Meier analysis (P = 0.035). **h** TUBA1C expression is an independent prognostic factor in LUAD by Multivariate Cox

The TCGA database was used to identify the differences in the mRNA levels of TUB1AC in normal tissues and LUAD tissues. The data obtained from 535 tumor tissues and 59 normal tissues was analyzed in this study. As shown in Fig. 1b, a boxplot was plotted using the data of expression of TUBA1C in normal and tumor tissues. The results indicate that the expression of TUBA1C in tumor tissues was significantly higher than in normal tissues (P = 8.913e-21). As shown in Fig. 1c, the Kaplan–Meier (KM) survival curve showed a high expression of TUBA1C in LUAD tissues, which indicated poor prognosis of LUAD. As shown in Fig. 1d, multivariable analysis was performed by adjusting for the following factors: age, stage, lymph node metastasis, and TUBA1C expression. Furthermore, boxplot, pathological stage plot, and survival curves were constructed by plotting the expression levels of TUBA1C with the help of GEPIA. As shown in Fig. 2, a high expression of TUBA1C was significantly associated with the following parameters: different disease states (Tumor or Normal) (P < 0.05), pathological stage (P = 1.82e-04), overall survival (P = 3.8e−05), and disease-free survival (P = 0.047). Immunohistochemical analysis of TUBA1C expression in 390 cases of lung adenocarcinoma and 60 cases of normal tissues is shown in Fig. 3a–f. From our data, there is no correlation between the level of TUBA1C expression and clinicopathological parameters of lung adenocarcinoma (Table 1). The KM curve analysis of TUBA1C shows the same results as TCGA data (Fig. 3g). Multivariable analysis demonstrated that only the TUBA1C expression was an independent prognostic factor in lung adenocarcinoma (Fig. 3h).

**Table 1:** The correlation of the level of TUBA1C expression with the clinical features of lung adenocarcinoma

Characteristics	TUBA1C (%)		p‐value
	Low	High	
n	149	241	
Age (years)			0.287
≤ 60	53 (35.6)	99 (41.1)	
> 60	96 (64.4)	142 (58.9)	
Sex			0.452
Female	60 (40.3)	87 (36.1)	
Male	89 (59.7)	154 (63.9)	
Smoke			0.511
No	123 (82.6)	192 (79.7)	
Yes	26 (17.4)	49 (20.3)	
Size			0.093
≤ 3 cm	92 (61.7)	127 (52.9)	
> 3 cm	57 (38.3)	113 (47.1)	
Histologic subtype			0.454
Lepidic	2 (1.3)	10 (4.1)	
Acinar	59 (39.6)	100 (41.5)	
Papillary	13 (8.7)	22 (9.1)	
Micropapillary	3 (2.0)	8 (3.3)	
Solid	72 (48.3)	101 (41.9)	
Lymph node metastasis			0.754
No	83 (55.7)	130 (53.9)	
Yes	66 (44.3)	111 (46.1)	
TNM			1
I + II	107 (71.8)	174 (72.2)	
III + IV	42 (28.2)	67 (27.8)	
VPI			0.758
No	131 (87.9)	209 (86.7)	
Yes	18 (12.1)	32 (13.3)	

### The relationship between the expression of TUBA1C and TIICs

**Fig.4:**
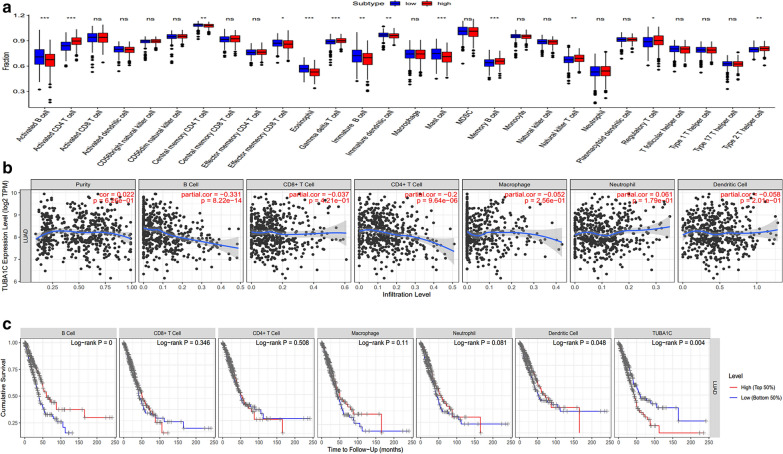
The relationship between the expression of TUBA1C and TIICs. **a** The results of the relative ratios of TIIC that were obtained using the CIBERSORT algorithm. The ratio of 28 immune cells in LUAD tissues in the TUBA1C high and low expression groups (*P < 0.05, **P < 0.01, ***P < 0.001). **b** Negative correlation exists between the TUBA1C expression level and infiltrating levels of B cell (r =  − 0.331, P = 8.22e−14) and CD4+ T cells (r =  − 0.2, P = 9.64e−06) in LUAD. **c** Cumulative survival is related to B cell and the expression of TUBA1C in LUAD. (The B cell and the expression of TUBA1C are factors related to the cumulative survival rate of LUAD over time)

Several evidences prove that the characteristics of TIICs were significantly correlated to the occurrence and development of tumor tissues [23–25]. Therefore, we explored whether the expression of TUBA1C was associated with TIICs in LUAD tissues. Based on the expression of TUBA1C, we segregated 497 samples into high and low expression groups. A total of 248 high expression samples and 249 low expression samples met the screening criteria. The two groups exhibited differences in the 28 proportions of immune cells, which were observed by downloading the gene expression source from an established and trusted computing resource (CIBERSORT, Newman et al., Stanford University, USA). Figure 4a displays the results of the 28 immune cell subsets. The expression of TUB1AC was significantly correlated to the following types of immune cells: activated B cell, activated CD4 T cell, central memory CD4 T cell, effector memory CD8 T cell, eosinophils, gamma-delta T cell, immature B cell, immature dendritic cell, mast cell, memory B cell, natural killer T cell, regulatory T cell, and type 2 T-helper cell. In particular, activated CD4 T cell, effector memory CD8 T cell, gamma-delta T cell, memory B cell, natural killer T cell, and regulatory T cell were present in higher proportions in the high expression group than in others. Moreover, activated B cell, central memory CD4 T cell, eosinophils, immature B cell, mast cell, and type 2 T -helper cell were also present in higher proportion in the high expression group (Fig. 4a). Meanwhile, we used TIMER and GEPIA databases to further investigate the relationship between TUBA1C and the diverse set of immune infiltrating cells. After adjusting the correlation coefficients according to purity, we found that the expression of TUBA1C was significantly correlated to T cells, B cells, natural killer cells, and neutrophils in LUAD tissues (Table 2). Furthermore, we used the GEPIA database to analyze the correlation between TUBA1C expression and the above-mentioned markers: T cell, B cell, natural killer cell, and neutrophils. The correlation results between TUBA1C and its related markers, namely, T cell, B cell, natural killer cell, and neutrophils were similar to those reported in TIMER database (Table 3).

**Table 2:** A Correlation analysis between TUBA1C and relate genes and markers of immune cells in TIMER

Description	Gene markers	LUAD			
		None		Purity	
		Cor	P	Cor	P
CD8+ T cell	CD8A	0.031	4.87e−01	0.037	4.18e−01
	CD8B	0.064	1.46e−01	0.064	1.53e−01
T cell (general)	CD2	− 0.122	5.43e−03	− 0.131	3.47e−03
	*CD3E*	*− 0.16*	*2.61e−04*	*− 0.178*	*6.96e−05*
B cell	*CD19*	*− 0.218*	*5.77e−07*	*− 0.247*	*2.88e−08*
	*CD79A*	*− 0.202*	*4.24e−06*	*− 0.221*	*7.46e−07*
Natural killer cell	KIR2DL1	− 0.016	7.21e−01	− 0.024	5.88e−01
	*KIR2DL3*	*0.13*	*3.16e−03*	*0.144*	*1.37e−03*
	*KIR2DL4*	*0.301*	*2.85e−12*	*0.312*	*1.39e−12*
	KIR3DL1	0.06	1.75e−01	0.065	1.51e−01
	KIR3DL2	0.048	2.82e−01	0.067	1.38e−01
	KIR3DL3	0.102	2e−02	0.112	1.29e−02
	*CD56*	*− 0.199*	*5.47e−06*	*− 0.205*	*4.40e−06*
Neutrophils	*CD66b*	*− 0.297*	*5.67e−12*	*− 0.298*	*1.53e−11*
	CD11b	− 0.075	8.78e−02	− 0.06	1.87e−01
	*CCR7*	*− 0.265*	*1.21e−09*	*− 0.291*	*4.62e−11*
Th1	T-bet	− 0.071	1.06e−01	− 0.079	7.84e−02
	STAT4	0.225	4.85e−02	0.173	1.44e−01
	TNF	0.243	3.08e−02	0.177	1.35e−01
Th2	GATA3	0.125	2.74e−01	0.168	1.56e−01
	STAT6	0.224	4.72e−02	0.22	6.16e−02
	STAT5A	0.259	2.17e−02	0.204	8.30e−02
	IL13	0.046	6.84e−01	0.134	2.58e−01
Tfh	BCL6	0.194	8.61e−02	0.246	3.61e−02
Th17	STAT3	0.293	8.93e−03	0.289	1.32e−02
	IL17A	0.199	7.89e−02	0.185	1.18e−01
T cell exhaustion	LAG3	0.067	5.55e−01	0.016	8.91e−01
	CTLA4	0.094	4.11e−01	0.028	8.16e−01
	TIM-3	0.185	1.02e−01	0.132	2.66e−01
Mast cells	TPSB2	0.051	6.58e−01	0.045	7.08e−01
	TPSAB1	0.031	7.84e−01	0.005	9.68e−01
	CPA3	0.142	2.13e−01	0.14	2.38e−01
	MS4A2	0.192	9.04e−02	0.176	1.35e−01
	HDC	0.062	5.87e−01	− 0.029	8.09e−01

**Table 3:** A Correlation analysis between TUBA1C and relate genes and markers of immune cells in GEPIA

Description	Gene markers	LUAD			
		Tumor		Normal	
		R	P	R	P
T cell (general)	*CD2*	− 0.15	*0.00096*	− 0.13	0.31
	*CD3E*	− 0.19	*2.7e−05*	− 0.057	0.67
B cell	*CD19*	− 0.27	*9.4e−10*	0.034	0.8
	*CD79A*	− 0.28	*4.3e−10*	0.056	0.67
Natural killer cell	KIR2DL1	− 0.008	0.86	0.12	0.38
	*KIR2DL3*	0.09	*0.048*	− 0.031	0.82
	*KIR2DL4*	0.26	*7.4e−09*	0.13	0.32
	KIR3DL1	0.014	0.76	− 0.05	0.71
	KIR3DL2	0.015	0.74	0.12	0.37
	KIR3DL3	0.076	0.093	0.076	0.57
	CD56	− 0.12	0.01	− 0.31	0.016
Neutrophils	*CD66b*	− 0.22	*6.6e−07*	0.06	0.65
	CD11b	− 0.039	0.4	− 0.0025	0.99
	*CCR7*	− 0.25	*3.2e−08*	0.18	0.18

### In the TIMER database, TUBA1C expression was associated with immune infiltration level in LUAD and cumulative survival in LUAD

In this study, TIICs played a decisive role in the prognosis and survival of LUAD patients [26]. Therefore, we used TIMER database to further explore the relationship between the prognosis and survival of infiltrating immune cells and TUBA1C expression in LUAD tissues. As shown in Fig. 4a, b negative correlation exists between TUBA1C expression levels and the infiltrating level of B cells (r =  − 0.331, P = 8.22e−14) and CD4+ T cells (r =  − 0.2, P = 9.64e−06). In LUAD tissues, TUBA1C expression was associated with poor prognosis and high immune infiltration. As shown in Fig. 4c, the infiltrating level of B cell and TUBA1C expression were the factors related to the cumulative survival rate of LUAD.

### The protein-coding genes of TUBA1C and its co-expression genes in LUAD

**Fig.5:**
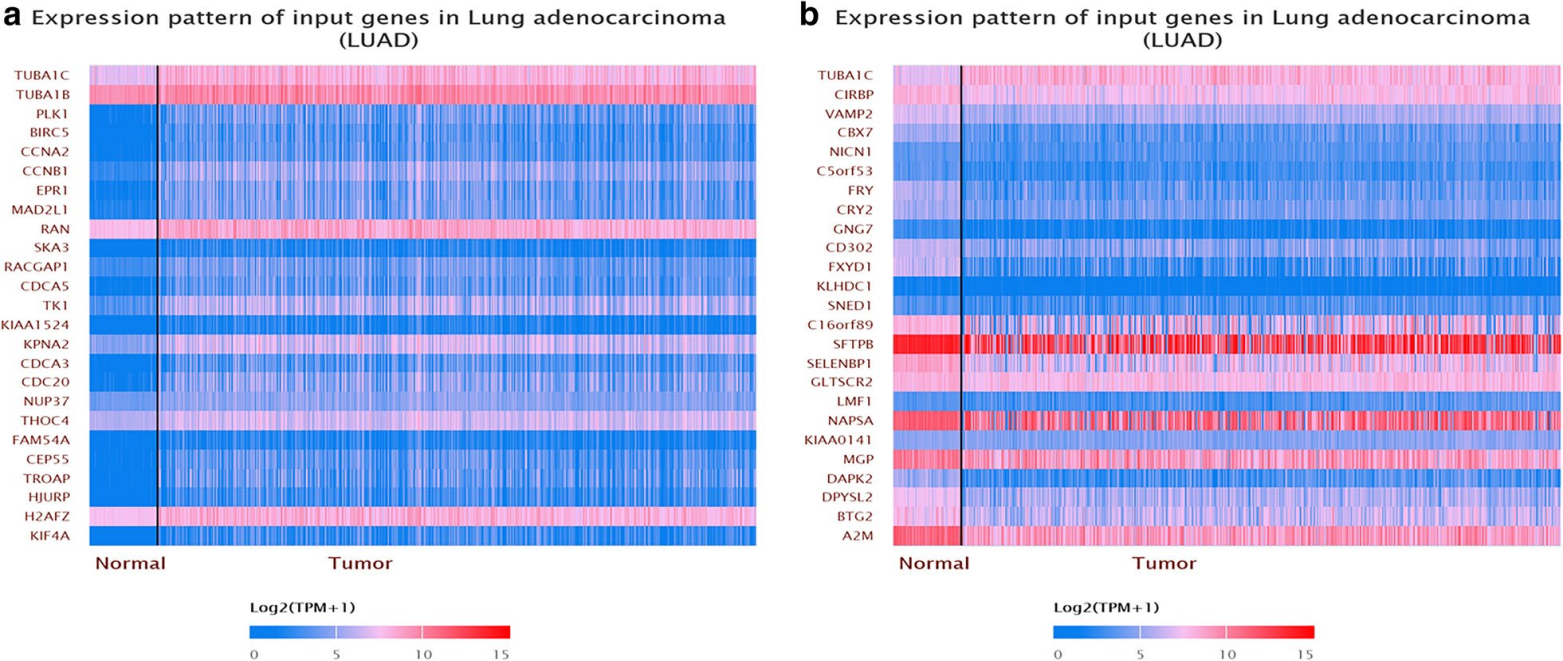
Genes correlated with TUBA1C in LUAD. **a** Genes positively correlated with TUBA1C in LUAD. **b** Genes negatively correlated with TUBA1C in LUAD

**Fig.6:**
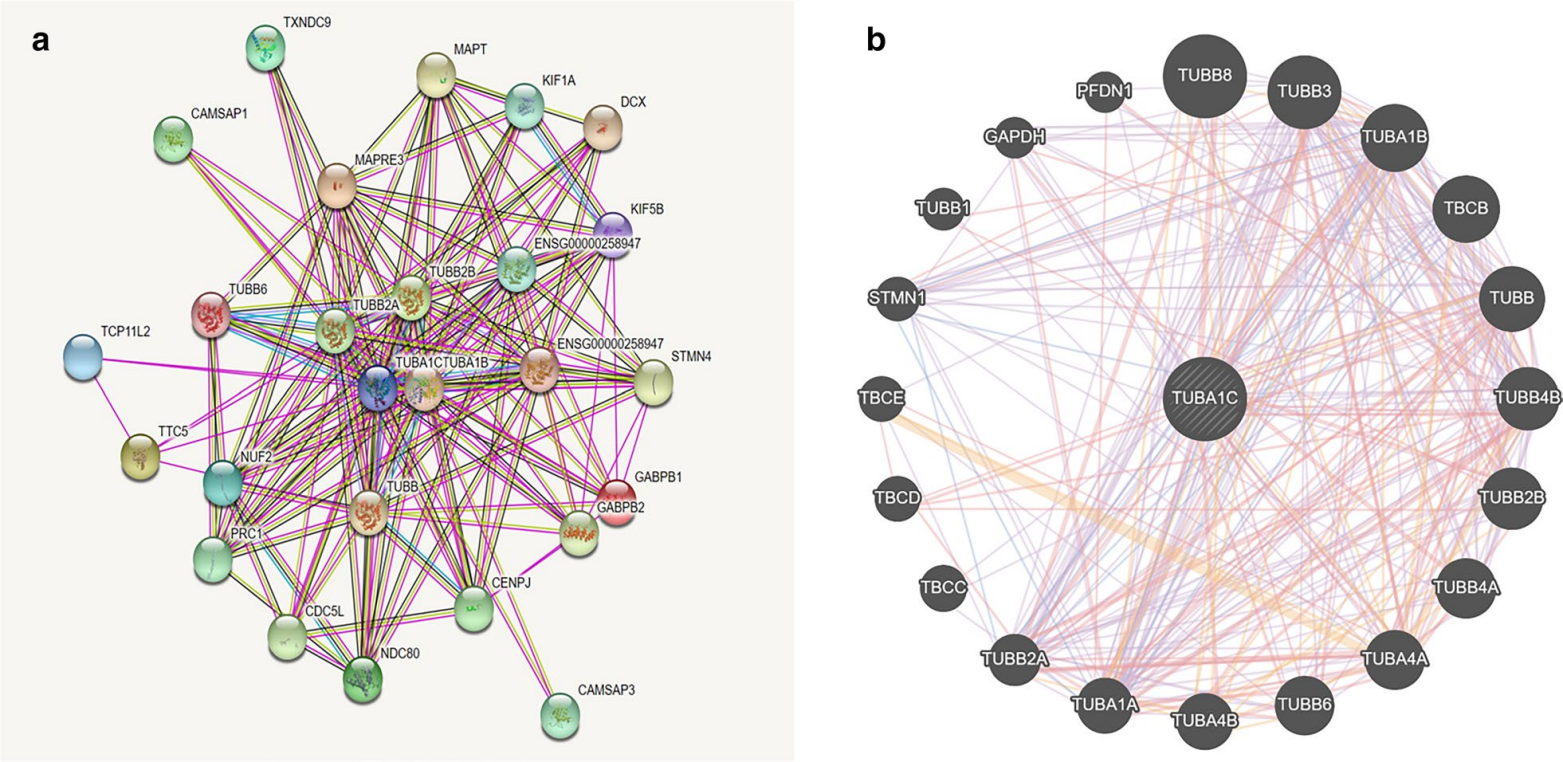
Protein–protein interaction network of TUBA1C. **a** Protein–protein interaction network of TUBA1C analyzed by STRING. **b** Protein–protein interaction network of TUBA1C analyzed by GeneMANIA

To investigate the potential molecular mechanisms through which TUBA1C elicits tumorigenesis in LUAD, we identified the protein-coding genes of TUBA1C and its co-expression genes in LUAD. As shown in Fig. 5, UALCAN database was used to identify genes that showed a positive and negative correlation with TUBA1C in LUAD tissues. The two-sided Pearson’s correlation coefficient analysis and z-test were performed by using R language, which is based on the gene expression data extracted from TCGA. The top 10 protein-coding genes that positively correlated with TUBA1C were as follows: TUBA1B, PLK1, BIRC5, CCNA2, CCNB1, EPR1, MAD2L1, RAN, SKA3, and PACGAP1. On the other hand, the top ten protein-coding genes that negatively correlated with TUBA1C were as follows: CIRBP, VAMP2, CBX7, NICN1, C5orf53, FRY, CRY2, GNG7, CD302, and FXYD1. Furthermore, STRING and GeneMANIA tools were used to analyze the interaction between TUBA1C and protein-coding genes mentioned earlier. Figure 6 illustrates the results of the analysis.

### Gene sets enriched in TUBA1C expression

**Fig.7:**
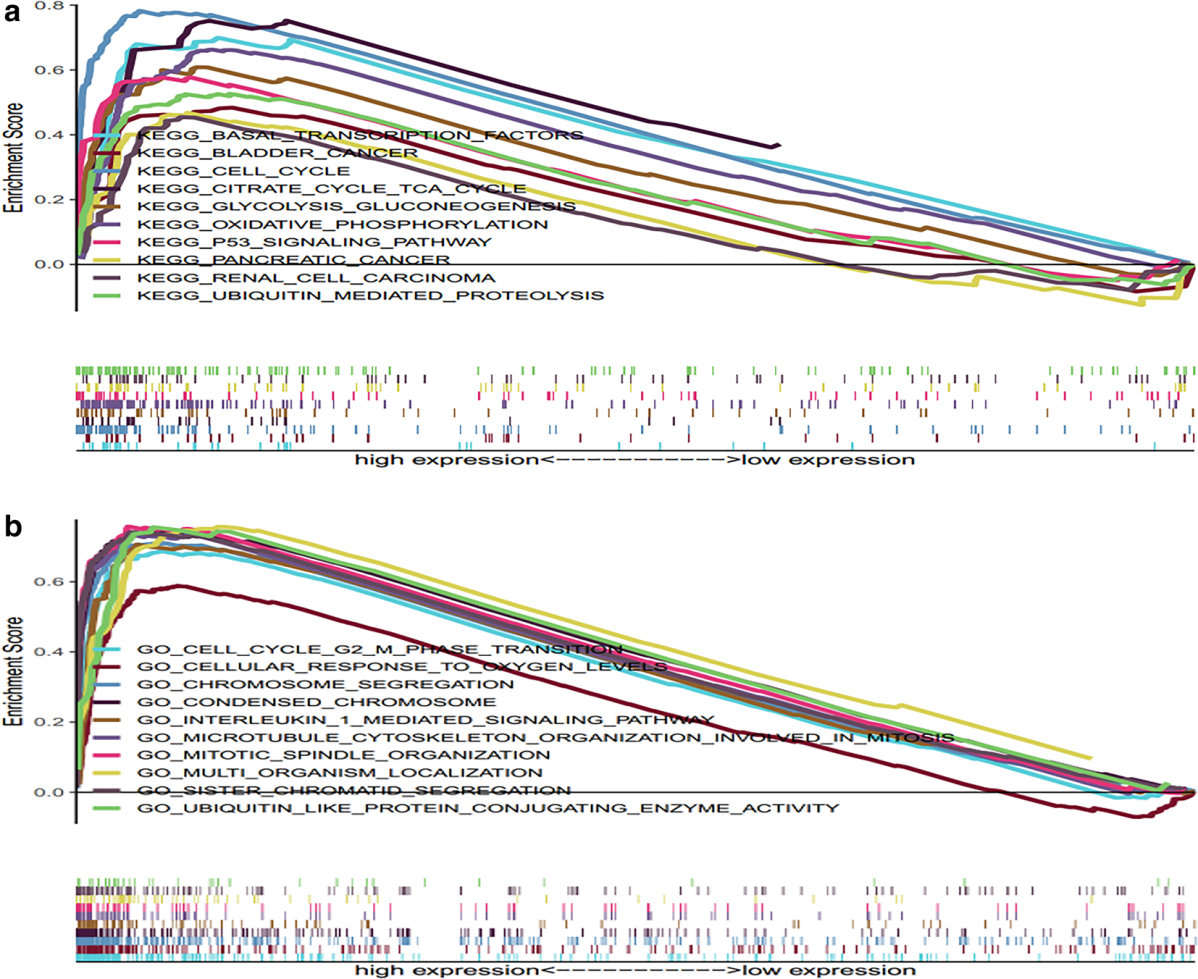
Enrichment plots from gene set enrichment analysis (GSEA). **a** GSEA results showing differential enrichment of genes in KEGG with high TUBA1C expression. **b** GSEA results showing differential enrichment of genes in GO with high TUBA1C expression

In this experiment, GO and KEGG pathway analysis were performed to determine the potential biological functions of TUBA1C. We observed a significant difference in the enrichment of GO and KEGG pathways, which were used to analyze samples that showed a high expression of TUBA1C (FDR < 0.050, P < 0.050). Table 4 presents 10 KEGG and GO pathways that were associated with a high expression of TUBA1C. As shown in Fig. 7a, the 10 KEGG pathways that were positively correlated to the high expression of TUBA1C were as follows: cell cycle, p53 signaling pathway, basal transcription factors, ubiquitin mediated proteolysis, glycolysis gluconeogenesis, citrate cycle TCA cycle, oxidative phosphorylation, pancreatic cancer, renal cell carcinoma, bladder cancer. As shown in Fig. 7b, GO analysis also revealed the following ten positively correlated categories: cell cycle G2/M phase transition, cellular response to oxygen levels, chromosome segregation, interleukin 1 mediated signaling pathway, multi-organism localization, condensed chromosome, ubiquitin like protein conjugating enzyme activity, microtubule cytoskeleton organization involved in mitosis, sister chromatid segregation, and mitotic spindle organization. These results indicate that the pathways of cell cycle, glycolysis, and gluconeogenesis are involved in the carcinogenesis of LUAD, which is strongly related to TUBA1C expression.

**Table 4:** Gene sets enriched in phenotype

Gene set name	NES	NOM p-val	FDR q-val
High expression			
KEGG_CELL_CYCLE	2.480884	0	0
KEGG_P53_SIGNALING_PATHWAY	2.169725	0	4.86e**−**04
KEGG_BASAL_TRANSCRIPTION_FACTORS	2.162689	0	8.37e**−**04
KEGG_UBIQUITIN_MEDIATED_PROTEOLYSIS	2.126961	0	9.34e**−**04
KEGG_GLYCOLYSIS_GLUCONEOGENESIS	2.00116	0	6.15e**−**03
KEGG_CITRATE_CYCLE_TCA_CYCLE	1.926811	0.004228	1.19e**−**02
KEGG_OXIDATIVE_PHOSPHORYLATION	1.815468	0.014706	2.55e**−**02
KEGG_PANCREATIC_CANCER	1.753528	0.012346	3.90e**−**02
KEGG_RENAL_CELL_CARCINOMA	1.732231	0.014553	4.46e**−**02
KEGG_BLADDER_CANCER	1.718563	0.011858	4.81e**−**02
GO_CELL_CYCLE_G2_M_PHASE_TRANSITION	2.451194	0	4.13e**−**04
GO_CELLULAR_RESPONSE_TO_OXYGEN_LEVELS	2.442309	0	2.06e**−**04
GO_CHROMOSOME_SEGREGATION	2.4354	0	1.65e**−**04
GO_INTERLEUKIN_1_MEDIATED_SIGNALING_PATHWAY	2.433667	0	1.50e**−**04
GO_MULTI_ORGANISM_LOCALIZATION	2.421357	0	2.64e**−**04
GO_CONDENSED_CHROMOSOME	2.415586	0	2.31e**−**04
GO_UBIQUITIN_LIKE_PROTEIN_CONJUGATING_ENZYME_ACTIVITY	2.389575	0	2.11e**−**04
GO_MICROTUBULE_CYTOSKELETON_ORGANIZATION_INVOLVED_IN_MITOSIS	2.380871	0	2.11e**−**04
GO_SISTER_CHROMATID_SEGREGATION	2.351018	0	2.04e**−**04
GO_MITOTIC_SPINDLE_ORGANIZATION	2.344327	0	1.88e**−**04

## Discussion

In molecular biology, TUBA1C is a kind of α-tubulin subtype that is related to microtubules. It is a multi-functional cytoskeleton protein, and it participates in the process of cell mitosis and cell division [27, 28]. Previous studies have reported that when the expression of TUBA1C is up-regulated, it significantly affects the growth and progression of tumor cells. This indicates that TUBA1C plays a pivotal role in the proliferation and cell cycle of various tumors [29, 30]. Recent studies have investigated the structure and function of microtubules (MT). These studies have reported its potential role in innate and adaptive immune systems [31]. Moreover, TIICs are found to be involved in the growth, invasion, and metastasis of lung cancer [32]. However, no previous study has reported about the relationship between TUBA1C expression and TIICs in LUAD tissues.

In this study, we investigated TUBA1C expression levels in various types of tumors, which were identified from the TCGA database by using TIMER algorithm. Compared to normal tissues, TUBA1C expression was found to be higher in the following types of tumors: BLCA, BRCA, CHOL, COAD, ESCA, HWSC, KIRC, KIRP, LIHC, LUAD, LUSC, PRAD, READ, STAD, THCA, and UCEC (Fig. 1a). The data of LUAD patients was acquired from TCGA database and used to estimate the prognosticative value of TUBA1C. Our main goal was to figure out whether TUBA1C can be used as a prognosticative biomarker. Furthermore, we found that the expression of TUBA1C was extremely higher in LUAD than in normal tissues. (Fig. 1b). As shown in Figs. 1c and 3g, the KM survival curve exhibited a high expression of TUBA1C in LUAD tissues, which indicated a poor prognosis of LUAD. Age, tumor grade, lymph node metastasis, and TUBA1C expression were considered as independent prognostic factors in multivariable analysis, which is illustrated as a forest boxplot in Fig. 1d. By using the GEPIA database, we found that TUBA1C expression was significantly related to the following parameters: different disease states (tumor or normal) (P < 0.05), pathological stage (P = 1.82e−04), overall survival (P = 3.8e−0.5), and disease-free survival (P = 0.047) (Fig. 2). By performing immunohistochemical analysis, we found that the expression level of TUBA1C was higher in tumor tissues than in normal tissues (Fig. 3a–f). From our data, there is no correlation between the level of TUBA1C expression and clinicopathological parameters of lung adenocarcinoma (Table 1). Multivariable analysis demonstrated that only the TUBA1C expression was an independent prognostic factor in lung adenocarcinoma (Fig. 3h). All these findings suggest that TUBA1C is a prognostic biomarker of LUAD.

In this study, we found that various immune infiltration levels of LUAD could induce different expression levels of TUBA1C. By using CIBERSORT algorithm, we found that TUBA1C expression is strongly associated with infiltration levels of following immune cells: activated B cell, activated CD4 T cell, central memory CD4 T cell, effector memory CD8 T cell, eosinophils, gamma-delta T cell, immature B cell, immature dendritic cell, mast cell, memory B cell, natural killer T cell, regulatory T cell and type 2 T-helper cell (Fig. 4a). Furthermore, TIMER and GEPIA databases showed how gene markers of different immune cells were related to TUBA1C expression. This may indicate that the tumor immune microenvironment (Tables 2, 3) of LUAD was regulated by TUBAC1. It is a well-known fact that T cells and B cells comprise about two-thirds of lung TIICs, while the remaining lung TIICs are composed of tumor-associated macrophages (TAMs) and a small number of infiltrating dendritic cells and natural killer cells (NK) [33]. Previous studies have thoroughly investigated the role of tumor- associated T cells in the development of lung cancer. These studies have reported that CD4 + Th1 cells and activated CD8 + T cells often elicited type I immune responses, which indicate a favorable prognosis of LUAD [34, 35]; however, Th2, Th17, and Foxp3 + regulatory T (Treg) cells were found to be associated with tumor progression and unfavorable prognosis [36]. In lung cancer patients, the tumor-infiltrating B cells have their own anti-tumor immunity. Moreover, the tumor-infiltrating B lymphocytes have been observed at all stages of lung cancer development. However, their morphology differed according to the stages of lung cancer and their histological subtypes. This indicates that B cells dominate during the progression of lung cancer [37–39]. Many studies have proved that the expression level of many genes is related to poor prognosis and TIICs. By analyzing the TIMER database, we found that the expression of TUBA1C is negatively correlated to B cells and CD4 + T cells. These results indicate a poor prognosis of LUAD (Fig. 4b, c).

Although TUBA1C is a tubulin, we still do not know the mechanism through it regulates LUAD. First, we analyzed the protein-coding genes related to TUBA1C and its co-expression genes in LADC tissues. The top 10 protein-coding genes that positively correlated with TUBA1C are as follows: TUBA1B, PLK1, BIRC5, CCNA2, CCNB1, EPR1, MAD2L1, RAN, SKA3, and PACGAP1. On the other hand, the top 10 negatively correlated genes are as follows: CIRBP, VAMP2, CBX7, NICN1, C5orf53, FRY, CRY2, GNG7, CD302, and FXYD1 (Fig. 5). Furthermore, STRING and Gene MANIA databases illustrated the protein interaction between TUBA1C and other partners (Fig. 6). The proteins related to TUBA1C perform following biological functions: they regulate the cell cycle, mitosis, DNA damage response, cell proliferation, and aging. Thereafter, GO and KEGG pathway analysis revealed that an up-regulated expression of TUBA1C is primarily related to cell cycle, p53 signaling pathway, glycolysis, and gluconeogenesis (Fig. 7, Table 4). Previous studies have also reported that TUBA1C is associated with cell proliferation, and it also regulates cell cycle in many types of cancers. Furthermore, the expression of TUBA1C was reported to be correlated with p53 expression in pancreatic ductal adenocarcinoma [12]. Previous studies have reported that tubulin regulates cell metabolism and glucose stress response, reducing the dependence of cells on glycolysis. These events ultimately promote cell survival in cancer patients [40]. These results are helpful to understand the biological role played by TUBA1C in the development of LUAD. In future clinical practice, the expression of TUBA1C in lung adenocarcinoma tissue may be used to predict the prognosis and the efficacy of immunotherapy of patients.

## Conclusion

In conclusion, this is the first report to prove that TUBA1C is a new marker of LUAD. This work further proved that TUBA1C played a pivotal role in the cell cycle and immune microenvironment of LUAD. By further understanding its range of functions, we can make TUBA1C an effective biomarker in the diagnosis and treatment of LUAD.

## Supplementary Information

**Additional file 1.** Supplementary data to this article.

## Data Availability

The datasets used and/or analyzed during the current study are available from the corresponding author upon reasonable request.

## Abbreviations

LUAD: Lung adenocarcinoma
TCGA: The Cancer Genome Atlas
GEPIA: Gene Expression Profiling Interactive Analysis
OS: Overall survival
DFS: Disease-free survival
TIICs: Tumor-infiltrating immune cells
GSEA: Gene set enrichment analysis
NSCLC: Non-small cell lung cancer
HCC: Hepatocellular carcinoma
IHC: Immunohistochemistry
GO: Gene ontology
KEGG: Kyoto Encyclopedia of Genes and Genomes
BLCA: Bladder urothelial carcinoma
BRCA: Breast invasive carcinoma
CHOL: Cholangiocarcinoma
COAD: Colon adenocarcinoma
ESCA: Esophageal carcinoma
HWSC: Head and neck squamous cell carcinoma
KIRC: Kidney renal clear cell carcinoma
LIHC: Liver hepatocellular carcinoma
LUSC: Lung squamous cell carcinoma
PRAD: Prostate adenocarcinoma
READ: Rectum adenocarcinoma
STAD: Stomach adenocarcinoma
THCA: Thyroid carcinoma
UCEC: Uterine corpus endometrial carcinoma
KM: Kaplan–Meier
TAMs: Tumor-associated macrophages
NK: Natural killer cells
